# Regulation of Fibrochondrogenesis of Mesenchymal Stem Cells in an Integrated Microfluidic Platform Embedded with Biomimetic Nanofibrous Scaffolds

**DOI:** 10.1371/journal.pone.0061283

**Published:** 2013-04-18

**Authors:** Weiliang Zhong, Weiguo Zhang, Shouyu Wang, Jianhua Qin

**Affiliations:** 1 Department of Orthopaedics, First Affiliated Hospital of Dalian Medical University, Dalian, P.R. China; 2 Department of Biotechnology, Dalian Institute of Chemical Physics, CAS, Dalian, P.R. China; University of Illinois, Urbana-Champaign, United States of America

## Abstract

In native fibrocartilage, mechanotransduction allows the cells to perceive the physical microenvironment not only through topographical cues from the extracellular matrix, but also through mechanical cues, such as interstitial flow. To create a microenvironment that simultaneously integrates nanotopography and flow stimulus, we developed a biomimetic microfluidic device embedded with aligned nanofibers to contain microchambers of different angles, which enabled the flow direction to form different angles with the fibers. Using this device, we investigated the effects of microfluidic and nanotopographical environment on the morphology and fibrochondrogenesis of mesenchymal stem cells (MSCs) and the involvement of RhoA/ROCK pathway and Yes-associated protein (YAP)/transcriptional co-activator with PDZ-binding motif (TAZ). The results showed that the flow direction perpendicular to aligned nanofibers was conducive to fibrochondrogenesis of MSCs. In addition, ROCK inhibitor and knockdown of YAP/TAZ disrupted fibrochondrogenic differentiation of MSCs. In conclusion, our data suggest the crucial role of mechanotransduction in regulating fibrochondrogenic differentiation of MSCs, which may be mediated by RhoA/ROCK pathway and YAP/TAZ.

## Introduction

Fibrocartilage, especially the meniscus of the knee and the annulus fibrosus of the intervertebral disc, have poor self-repair capability, particularly in avascular regions [1]. In the past decade, mesenchymal stem cells (MSCs) have shown promise for regenerative medicine, especially tissue engineering, due to their capacity of self-renewal, proliferation, and pluripotency. The differentiation of MSCs can be directed toward many tissue-specific cell types by modulating the extracellular, biochemical, and biophysical microenvironment [2]–[4]. Accordingly, MSC-based tissue engineering offers a promising remedy for injured fibrocartilage. Several studies have shown that mechanical or topographical cues exert considerable influence on MSC fibrochondrogenic differentiation [5]–[7].

Nanofibrous scaffolds, formed by the electrospinning technique, have been widely employed for tissue engineering to mimic the native extracellular matrix (ECM). These scaffolds provide a biomimetic nanotopographical microenvironment capable of modulating cell morphology, differentiation, phenotype, and cytoskeletal organization by virtue of their nano-scale features [8]. It is well known that fibrocartilage is highly ordered and densely packed with locally aligned collagen fibers [9]. Therefore, mimicking the features of localized ECM by using aligned nanofibers may be conducive to direct tissue growth. Indeed, aligned nanofibrous scaffolds can augment matrix content and serve as instructive topographical cues for stem cell fibrochondrogenic differentiation [10].

The native mechanical milieu of fibrochondrocytes is complex, which involves tension, compression, shear, interstitial fluid flow, and hydrostatic pressure [11]. Accordingly, many recent studies have focused on MSC fibrochondrogenesis regulated by mechanical cues [12]–[15]. However, the effects of fluid flow, especially oscillatory fluid flow, on MSC fibrochondrogenic differentiation have not yet been well established. In native fibrocartilage, cell metabolism and nutrient transport are mainly regulated by the interstitial fluid flow through the matrix, which is driven by dynamic mechanical loading [16]. Oscillatory fluid flow is a significant mechanical stimulus that could be used to mimic the extracellular microenvironment *in vitro*. However, it remains unclear whether the direction of oscillatory flow acting on aligned nanofibrous scaffolds will modulate MSC fibrochondrogenic fate.

The regulation of MSC morphology and differentiation by mechanical or topographical niche depends on the ability of the cells to perceive and translate these stimuli into biochemical signals via mechanotransduction mechanisms [17]. The RhoA/ROCK pathway, which mediates mechanical cues, has been confirmed to play a key role in regulating cytoskeletal dynamics and stem cell differentiation [18]–[20]. Cell responses to fluid flow-induced and topography-induced mechanical signals are regulated mainly via the RhoA/ROCK pathway [21], [22]. The transcription coactivators Yes-associated protein (YAP) and transcriptional co-activator with PDZ-binding motif (TAZ) play key roles in cell proliferation, survival, differentiation, tissue regeneration, and organ size determination. Recent studies suggest the role of YAP/TAZ in the nuclear transduction of mechanical and cytoskeletal signals [23], [24]. The activity of YAP/TAZ to acts as a sensor and a mediator of mechanical and cytoskeletal signals depends on Rho GTPase activity [25]. It has been reported that the active state of the intracellular mediator of mechanical cues has entirely different effects on stem cell fate. For instance, the activation of RhoA/ROCK pathway or YAP/TAZ directed commitment of MSCs toward an osteogenic fate while inactive RhoA/ROCK pathway or inactive YAP/TAZ promoted adipogenic fate [7], [25]. Moreover, the connection between ROCK signaling and chondrogenic differentiation is dependent on the cellular context [20], [26]. Of note, ROCK can enhance the activity of Sox9, which is an essential transcription factor for chondrogenesis [27]. Nonetheless, the role of YAP/TAZ in fibrochondrogenic differentiation of MSCs is not well understood. In addition, it remains unclear whether YAP/TAZ plays similar role to RhoA/ROCK pathway in fibrochondrogenesis in a mechanically and nanotopographically biomimetic microenvironment.

To date, microfluidic devices have been increasingly employed to investigate cellular biomechanics or mimic native tissue architecture [28], [29]. These microdevices may save time and resources by increasing experimental throughput and minimizing the experimental platform. Nevertheless, it is still challenging to integrate nanostructures into a microfluidic platform. In this study, to create a microenvironment that incorporates nanotopography and flow stimulus simultaneously, we integrated a microfluidic device containing microchambers of multiple angles with aligned nanofibrous substrates. This design enabled the fluid flow direction to form different angles with the aligned nanofibers. In addition, we attempted to mimic a native microenvironment *in vitro*, using aligned nanofibers for topographical cues and fluid flow for extrinsic mechanical cues. We then investigated MSC morphology and fibrochondrogenesis within our manufactured biomimetic microenvironment. Furthermore, we explored the role of RhoA/ROCK pathway and YAP/TAZ in MSC fibrochondrogenic differentiation.

## Materials and Methods

### Preparation of Electrospun Fibers

Poly lactic-co-glycolic acid (PLGA; viscosity 1.64 dL/g; Changchun Sibobiomaterials, China) was dissolved in trifluoroethanol at a concentration of 15% (w/v) by stirring for 3–4 h. The electrospinning apparatus used in this study was operated according to procedures previously described [30]. Briefly, one 1 mL syringe fitted with a stainless-steel blunt needle was connected to a high-voltage power supply and placed perpendicularly to the collector with a distance of 15 cm. The electrospinning process was carried out with the following parameters: applied voltage, 11 kV; solution feed rate, 0.4 mL/h; rotor speed, 900 rpm for aligned nanofibers. After fabrication, each fiber film was transferred onto a clean glass slide and dried overnight under vacuum at 50°C.

### Scanning Electron Microscopy (SEM)

The electrospun samples were mounted on aluminum stumps and sputter-coated with gold. They were then observed under SEM (JSM-6360 LV, Japan). Both the diameter and alignment of nanofibers were analyzed with Image J software (NIH, USA).

### Design and Fabrication of Microfluidic Device Embedded with Nanofibers

**Figure 1:**
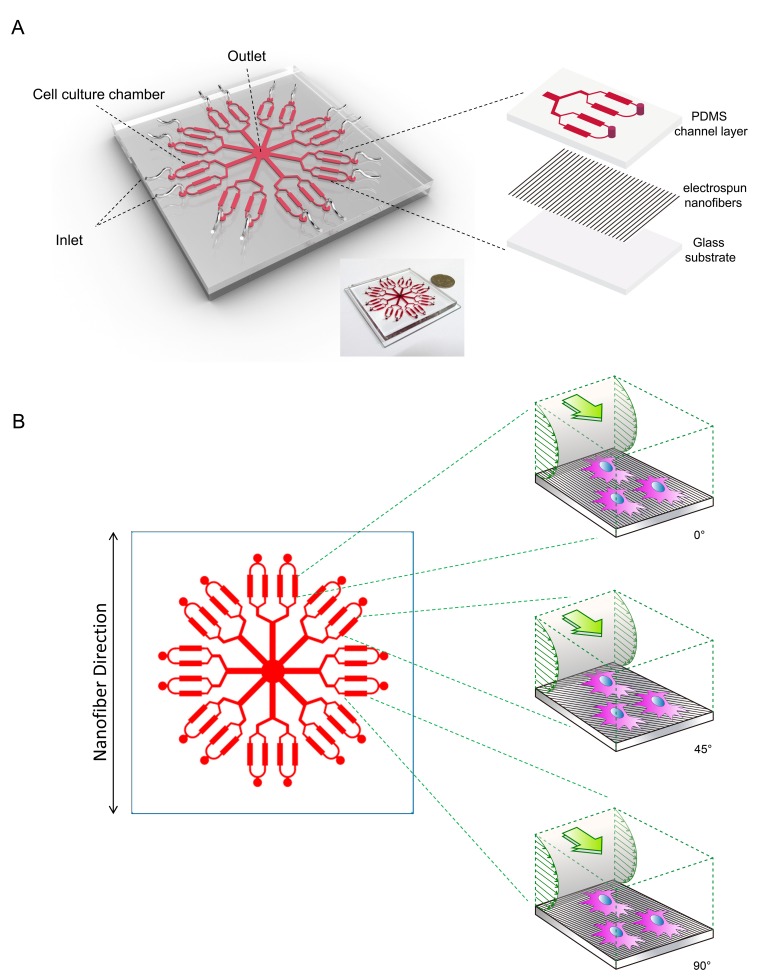
Microfluidic-nanofiber device design. A: Schematic diagram of the integrated microfluidic device embedded with aligned nanofibrous scaffolds used for dynamic culture. B: The layout of microfluidic networks enable the fluid flow direction to form different angles (0°, 45°, 90°) with the aligned nanofibers simultaneously.

The microfluidic device design was shown in Fig. 1. The device had 16 inlets, 1 outlet, and 32 microchambers. Both microchannels and microchambers were 100 µm in height, and each microchamber was 1.8 mm in width and 8 mm in length. The device consisted of 3 layers: the upper polydimethylsiloxane (PDMS) layer for microchannel networks, the middle aligned electrospun nanofiber layer, and the lower substrate glass layer (Fig. 1A). The microchambers and main microchannels were designed to make an angle of 0°, 45° (135°), and 90° with the nanofiber alignment direction. The inlets and outlet of the device were connected to a flow-driving system comprising a multi-channel peristaltic pump (Longer Pump, China) and a culture medium reservoir. Based on the nanofiber direction relative to the flow direction in the microchamber, the relationships between them were classified into three cases: parallel, 45° (135°), and perpendicular (Fig. 1B).

**Figure 2:**
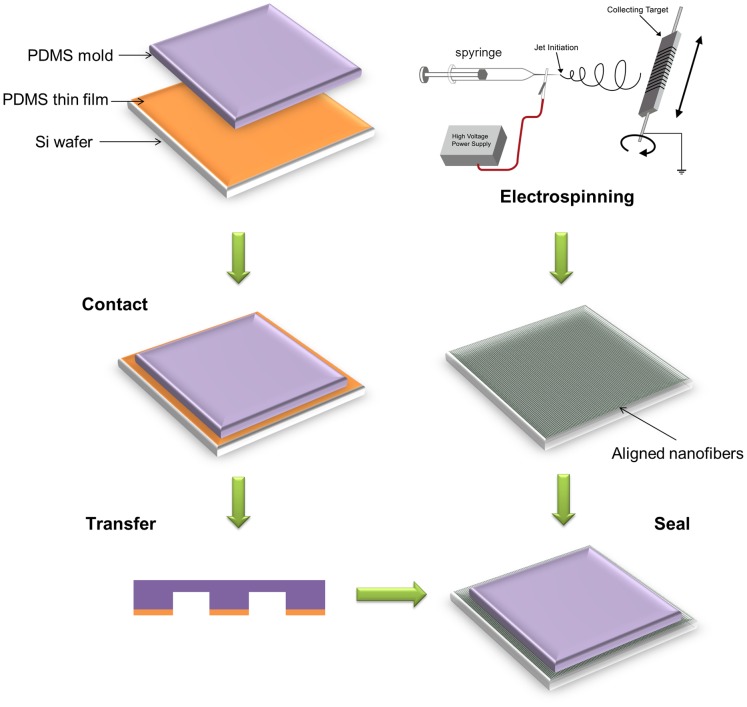
Schematic of the fabrication process of the microfluidic-nanofiber device.

The PDMS (Sylgard 184, Dow Corning, USA) replica of the microchannel structures was fabricated using microfabrication techniques involving SU-8 (Microchem, USA) photolithography and PDMS soft lithography [31]. Briefly, the mask was designed in AutoCAD 2007 (Autodesk) and printed on transparencies with 4000 dpi resolution. The transparency mask was used in 1∶1 contact photolithography with SU-8 photoresist to yield a negative master which was formed from a relief of photoresist on a silicon wafer. The PDMS prepolymer mixed with curing agent (Sylgard 184 silicone elastomer kit, Dow Corning, USA) at 10∶1 (w/w) was degassed to remove any bubbles, poured over the patterned wafer to completely cover the pattern and placed at 80°C for 1 h in the oven to cure. The PDMS replica of the network design was cut out and punched out in the position of the inlet and outlet for the fluid using sharpened blunt-tip needles. A mixture of PDMS resin and curing agent (10∶1) was diluted with methylbenzene (2∶3). One milliliter of the solution was spin-coated onto a Si wafer at 2000 rpm for 1 min. Replica molding of PDMS was brought into contact with the PDMS thin film, as shown in Fig. 2. After 30 sec, the PDMS mold was then taken off from the wafer and brought into contact with the aligned nanofiber sheet on the glass substrate and placed at 80°C for 1 h in a vacuum oven to complete the assembly (Fig. 2). PDMS diluted with methylbenzene could penetrate into the porous nanofiber sheets quickly, and then bond with the surrounding nanofibers and glass substrate at the bottom by consolidation. The PLGA nanofibers embedded in the microchambers of the microfluidic device with continuous perfusion of medium were observed under a microscope perfusion.

### Isolation and Culture of Rat MSCs

Animal care and treatment procedures were strictly conducted in accordance with institutional guidelines of national and international laws and policies. All experimental procedures were approved by the Committee on Animal Use and Care of Dalian Medical University. Primary MSCs were isolated from the bilateral femurs and tibias of young adult male Sprague-Dawley rats with the weight of 80–120 g. The distal ends of the bone were cut open and the marrow cavities were lavaged with sterile phosphate-buffered saline (PBS). The cells were resuspended in low glucose DMEM (GIBCO Invitrogen, USA) containing 10% FBS (Hyclone, USA) and 100 units/mL penicillin-streptomycin, and then plated into 25 cm^2^ flasks. After 48 h incubation at 37°C in 5% CO_2_, the medium was changed to remove non-adherent cells. At two passages, MSC cultures were devoid of non-adhering cells and used in the following experiments.

### Characterization of Isolated MSCs

To confirm the undifferentiated state of the isolated rMSCs, cell surface antigen markers, including CD90, CD29, and CD45 were probed via flow cytometry (FCM) analysis with the corresponding antibodies (BD Biosciences) [32], . Multi-lineage differentiation experiments, which included adipogenesis and osteogenesis, were performed to characterize the pluripotency of MSCs, as previously described [2]. Briefly, MSCs were incubated in a six-well plate at a density of 5×10^4^ cells/well with Rat Adipogenic and Osteogenic Differentiation Medium (Cyagen Biosciences, USA) for adipogenesis and osteogenesis induction, respectively. Adipogenic differentiation was assessed through oil red O staining after a week of culture. Osteogenic differentiation was confirmed through alkaline phosphatase (ALP) staining after two weeks of culture.

### Cell Culture in Microfluidic-nanofiber Device

The microfluidic device was sterilized under UV light for overnight. The microchambers were coated with 100 µg/mL fibronectin (Sigma, USA) for 1 h at room temperature, and then washed with PBS. MSC suspension of 5×10^6^ cells/mL was injected into the chambers from the inlet and incubated at 37°C for 12 h to allow cell attachment. Next the inlets of the device were connected to a multi-channel peristaltic pump and the outlet was connected to a reservoir for oscillating perfusion culture. The dynamic flow profile was sinusoidal at a frequency of 0.1 Hz. With a low height to width ratio, the microchamber was treated as a parallel plate system. The transient shear stress was approximated by equation 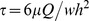 [34], [35], where 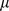 is the dynamic viscosity (1×10^−3^ Pa s), *Q* is the flow rate, and 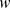 and 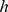 are the microchamber width and height, respectively. The peak fluid shear stress near the surface was calculated to be ±0.01 dyne/cm^2^ and ±1 dyne/cm^2^ for the flow rates of 0.36 and 36 µL/min, respectively. For fibrochondrogenesis, rat chondrogenic differentiation medium (Cyagen Biosciences, USA) was used as the perfusion medium for 4 weeks of perfusion culture. Static culture was used as a control group, the differentiation medium was replaced twice a week for 4 weeks.

### ROCK Inhibition and YAP/TAZ Knockdown

MSCs were exposed to ROCK inhibitor Y-27632 dihydrochloride (10 µM) (Sigma, USA) for 1 h before perfusion culture. Next the chondrogenic differentiation medium was supplemented with relatively low dose of Y-27632 dihydrochloride (5 µM) for both perfusion culture and static culture.

In order to inhibit YAP/TAZ activity, MSCs were transfected with the indicated siRNAs (siControl; siYAP; siTAZ; and siYAP/TAZ Santa Cruz, USA) by using siRNA transfection reagent (Santa Cruz, USA) according to manufacturer’s protocol. The specific depletion of endogenous YAP and TAZ was confirmed by Western blot analysis using YAP or TAZ antibody (Santa Cruz, USA).

### Immunofluorescence Staining and Image Analysis

Samples in the device were washed with PBS, fixed with 4% paraformaldehyde at room temperature for 15 min, and permeabilized with 0.1% Triton X-100 for 10 min. After washing with PBS 3 times, the samples were blocked with normal goat serum at room temperature for 30 min, then incubated with primary antibodies against F-Actin (Bioss, China), Collagen I (Sigma, USA), Collagen II (Sigma, USA) at 4°C overnight; and further incubated with FITC-conjugated goat anti-rabbit IgG or TRITC-conjugated goat anti-rabbit secondary antibodies (Zhongshan, China) at room temperature for 1 h. Nuclear staining was carried out using 4,6-diamino-2-phenyl indole (DAPI) (Invitrogen, USA) for 10 min. After staining, the devices were washed with PBS 2–3 times and imaged using fluorescence microscopy (Olympus IX71). The fluorescence images were analyzed with Image-Pro Plus 6.0 to measure the area and perimeter of individual cells, which were used to calculate shape index (SI). The SI is a nondimensional number defined as: 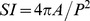, where A is cell area and P is cell perimeter. It ranges from 0 (a straight line) to 1 (a perfect circle), which indicates the degree of elongation of a cell [34]. The major and minor axes of the nuclei were measured using Image J software. The nuclear aspect ratio (NAR) was calculated as the ratio of the major axis to the minor axis as previously described [36].

### Alcian Blue Assay

Samples were stained for proteoglycans as previously described [37]. After being fixed for 15 min with 4% glutaraldehyde at room temperature, the samples were rinsed with 0.1 N HCl to decrease the pH to 1.0, stained 30 min with 1% Alcian blue solution (Sigma, USA), and rinsed twice with 0.1 N HCl to remove nonspecific staining.

### Quantitative Real-time RT-PCR

Real-time RT-PCR was used to analyze the mRNA expression of Sox-9, Runx-2, aggrecan, collagen I, and collagen II. Briefly, total RNAs were extracted and reverse transcribed into cDNA using Cell Amp® Whole Transcriptome Amplification Kit (Real Time) Ver.2 (Takara). Real-time RT-PCR was performed with an Mx3000P QPCR system (Agilent Technologies, USA) using SYBR® *Premix Ex Taq*™ II (Takara). β-actin was used as the internal control gene to normalize the quantities of target gene expressions. Thermocycling conditions were as follows: 95°C for 30 seconds 40 cycles of denaturation (95°C, 5 s), annealing (60°C, 30 s) and extension (72°C, 30 s). The primers used by real-time PCR are listed in Table 1. The data was analyzed using the 2^−ΔΔCt^ method.

**Table 1:** Sequences of primers used in real-time RT-PCR.

Gene	PCR primer sequences (forward and reverse)	GenBank Accession
Aggrecan	Forward 5′-GGCCTTCCCTCTGGATTTAG-3′	NM_022190
	Reverse 5′-CCGCACTACTGTCCAAC-3′	
Collagen II	Forward 5′-CCCCTGCAGTACATGCGG-3′	NM_012929
	Reverse 5′-CTCGACGTCATGCTGTCTCAAG-3′	
Collagen I	Forward 5′-TCCAGGGCTCCAACGAGA-3′	NM_053304
	Reverse 5′-CTGTAGGTGAATCCACTGTTGC-3′	
Runx2	Forward 5′-TAAAGTGACAGTGGACGGTCCC-3′	NM_053470
	Reverse 5′- TGCGCCCTAAATCACTGAGG -3′	
Sox9	Forward 5′-CTGAAGGGCTACGACTGGAC-3′	RGD_620474
	Reverse 5′-TACTGGTCTGCCAGCTTCCT -3′	
β-Actin	Forward 5′- AGGGAAATCGTGCGTGAC-3′	NM_031144
	Reverse 5′- CGCTCATTGCCGATAGTG-3′	

### Statistical Analysis

All the experiments were performed using at least three biological replicates in triplicate of the device and cells. Difference among three groups was analyzed by using one-way analysis of variance (ANOVA). Comparison between two groups was done using the two-sample t-test.

## Results

### Characterization of PLGA Electrospun Meshes

**Figure 3:**
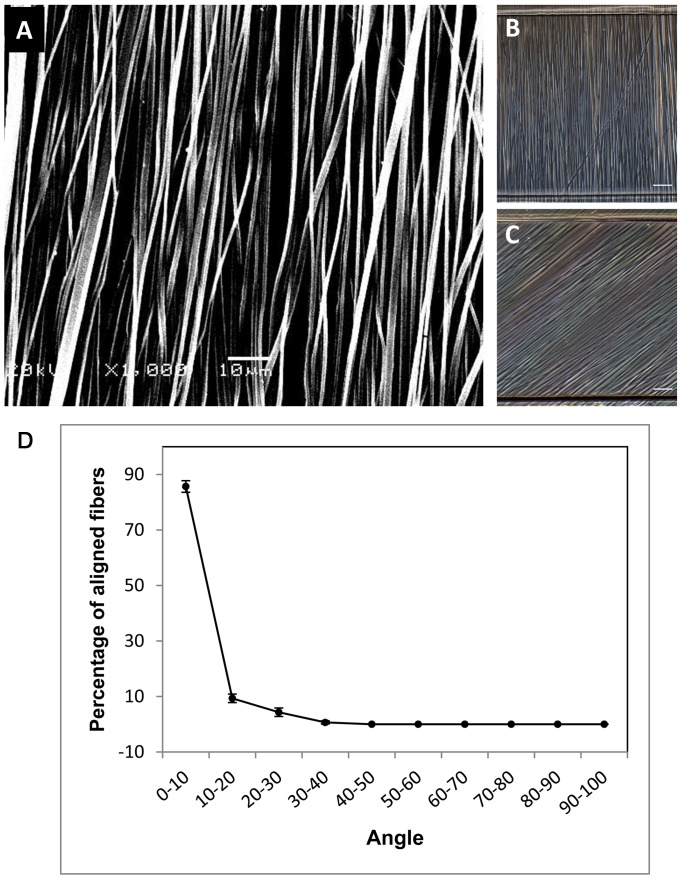
Characterization of PLGA meshes with aligned electrospun nanofibers. A: SEM image of PLGA nanofibers. Scale bar: 10 µm. B and C: The microscope images of PLGA nanofibers embedded in the perfusion microchambers of the microfluidic device. Scale bar: 50 µm. D: Graph depicting the percentage of aligned fibers in the meshes. Data are presented as mean ± SD.

SEM images showed that the uniform PLGA fibers without bead defects were formed by electrospinning (Fig. 3A). The average diameter of the aligned fibers was 809±101 nm. After being combined with the microfluidic device, the PLGA nanofibers in the microchambers with continuous perfusion of medium were still uniform and aligned (Fig. 3B, C). In addition, more than 80% of the fibers were aligned within 10° when they were deposited on a collector rotating at a high speed (Fig. 3D).

### Characterization of MSCs

**Figure 4:**
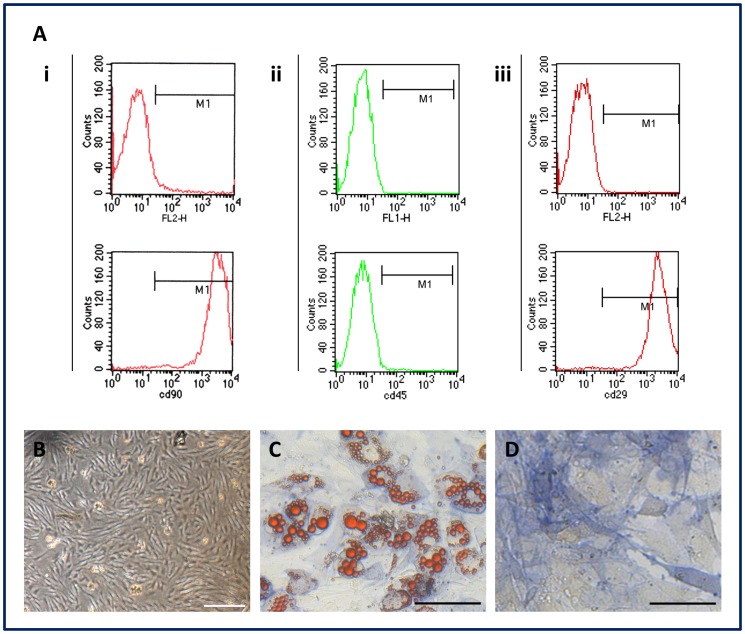
Characterization of MSCs. A: Flow cytometry analysis showed that the cells were positive for i) CD90 (99.18%±1.02%) and iii) CD29 (99.17%±1.07%), negative for the hematopoietic marker ii) CD45 (0.76%±0.25%). B: Cells at passage 2 exhibited spindle-shaped morphology and homogeneous phenotype. C: Oil red O staining showed intracellular lipid droplets after induction of adipogenic differentiation. D: Alkaline phosphatase was detected in the cytoplasm after induction of osteogenic differentiation. Scale bar: 100 µm.

The cultured MSCs showed a fibroblast-like, spindle-shaped morphology and a homogeneous phenotype (Fig. 4B). FCM analysis showed that the cells at passage 2 were uniformly positive for CD29 (99.17%±1.07%) and CD90 (99.18%±1.02%), but negative for the hematopoietic marker CD45 (0.76%±0.25%) (Fig. 4Ai–iii). The multi-lineage differentiation experiments showed that MSCs were successfully differentiated into adipocytes and osteocytes. Intracellular lipid droplets were observed after induction of adipogenesis (Fig. 4C), while alkaline phosphatase was detected in the cytoplasm after induction of osteogenesis (Fig. 4D). These results demonstrated that the isolated MSCs were proliferative and pluripotent.

### Morphological Response of MSCs to Flow with Aligned Nanofibers at Multiple Angles

**Figure 5:**
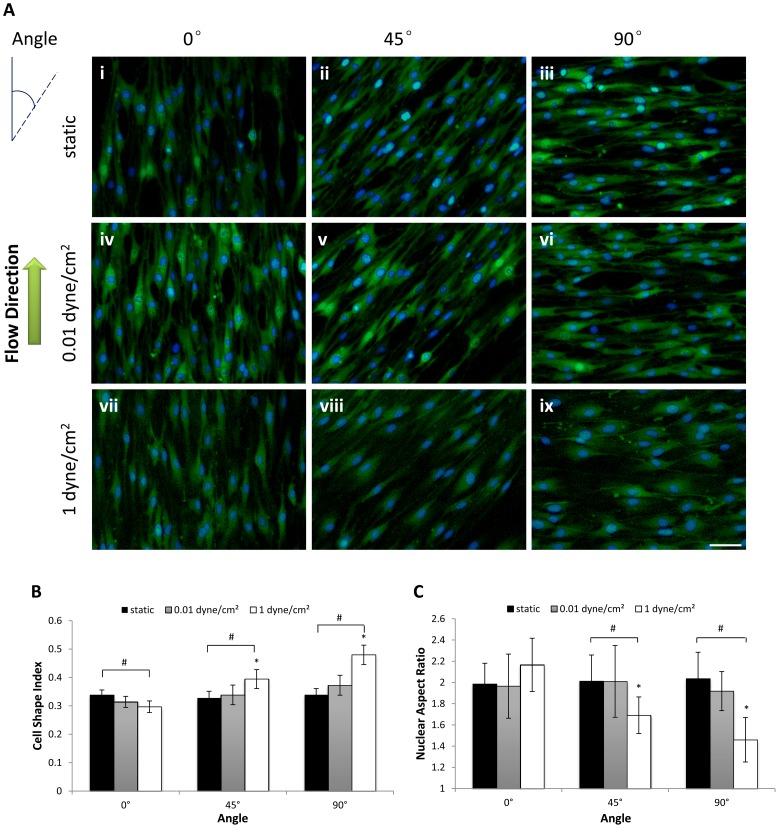
Morphological response of MSCs to flow with aligned nanofibers at multiple angles. A: Cytoskeletal and nuclei morphology of rMSCs cultured in microfluidic-nanofiber device for 24 h. The flow direction coinciding with the fiber direction was defined as 0°. Green = f-actin. Blue = nuclei. Scale bar: 50 µm. B: Variation of shape index (SI) under each condition. C: Variation of nuclear aspect ratio (NAR) under each condition. Data are presented as mean ± SD of n = 30 cell bodies and nuclei measured for three different batches of devices for each condition. *p<0.01 vs. 0°. ^#^ *p*<0.01 vs. static.

To determine the response of MSCs to flow with aligned nanofibers at multiple angles, cells were cultured in the device under both static and dynamic conditions. As shown in Fig. 5A, MSCs exhibited different cytoskeletal morphology under various conditions. When MSCs were seeded and grown on the PLGA meshes, their extensions were affected by the underlying fiber orientation. The cells had an elongated shape along the aligned fibers (Fig. 5Ai–iii). The cells were then subjected to oscillatory fluid flow that yielded peak shear stresses of 1 dyne/cm^2^ and 0.01 dyne/cm^2^ for 24 h. When the flow direction was parallel to the nanofiber direction (0°), a synergic effect of topography and flow was observed. With the increasing magnitude of fluid shear stress, the cells had a more elongated shape along the aligned fibers (Fig. 5Aiv and vii) than the static condition (Fig. 5Ai). However, when the flow direction was perpendicular to the nanofiber direction (90°) at 1 dyne/cm^2^, the cells spread more in the flow direction and showed a relatively more rounded or polygonal shape, but still steered along the nanofiber direction (Fig. 5Aix).

The cell and nuclear morphology was quantitatively evaluated in terms of SI (Fig. 5B) and NAR (Fig. 5C), respectively. When the flow direction coincided with the fiber direction (0°), SI was significantly lower at 1 dyne/cm^2^ relative to the static condition. When flow direction and fiber direction formed an angle of 45° or 90°, SI was significantly higher at 1 dyne/cm^2^ relative to the static condition. Meanwhile, under 45° or 90°, NAR was significantly lower at 1 dyne/cm^2^. Under the same perfusion conditions, SI and NAR under 45° and 90° were significantly higher and lower, respectively, than that of the 0° condition at 1 dyne/cm^2^. Both SI and NAR at 0.01 dyne/cm^2^ did not differ significantly under the 45° or 90° condition.

### Effects of Flow Stimulus at Different Angles with Aligned Nanofibers on MSC Fibrochondrogenesis

**Figure 6:**
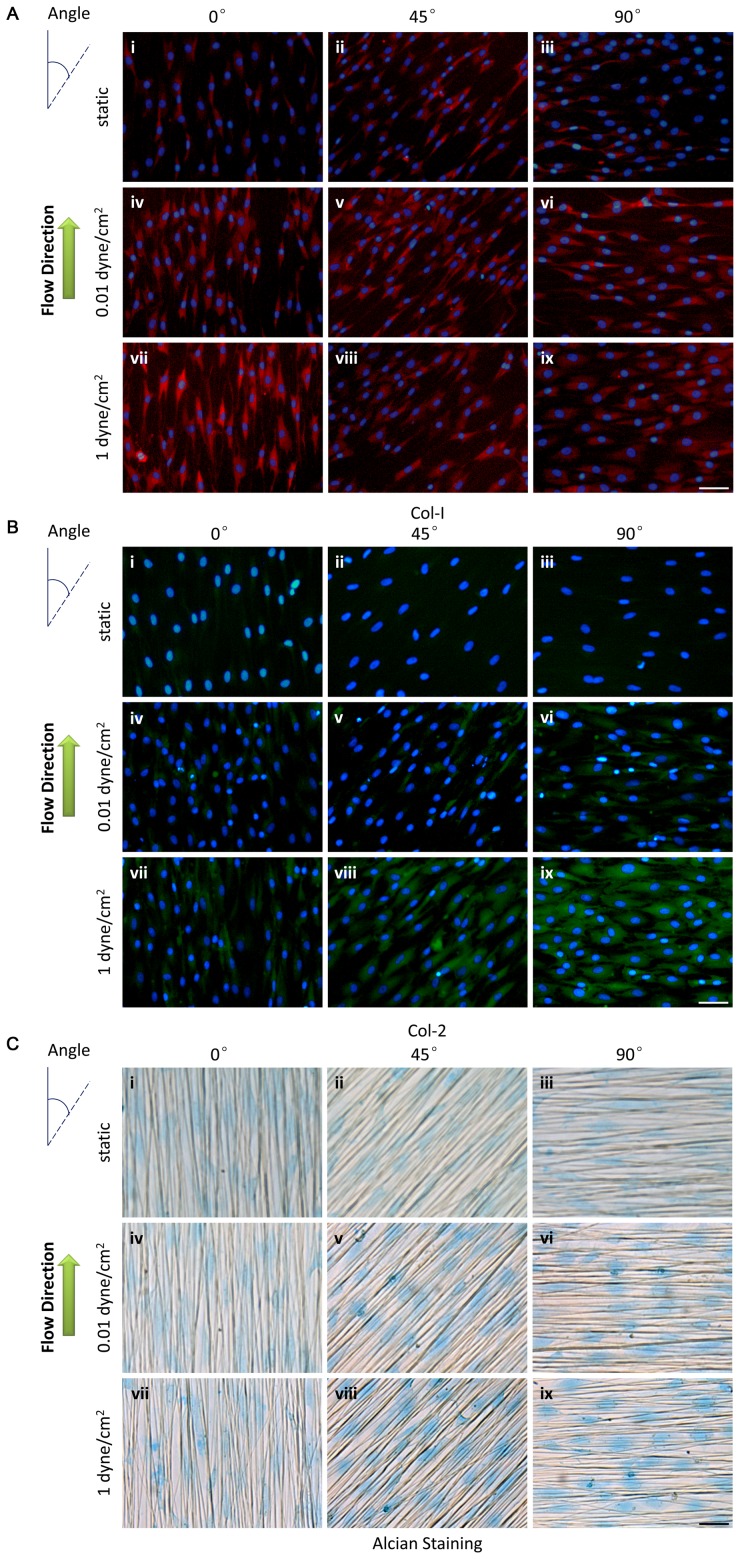
The expression of fibrochondrogenic markers after 4 weeks of induced differentiation of MSCs under flow stimulus at different angles with aligned nanofibers. A: Immunofluorescence staining for collagen I (red) and DAPI (blue). B: Immunofluorescence staining for collagen II (green) and DAPI (blue). C: Alcian blue staining for proteoglycans. Scale bar: 50 µm.

In order to assess the combined effect of flow and nanotopographical cues on fibrochondrogenesis of MSCs, the expression of fibrochondrogenic markers and fibrochondrogenesis-related genes were examined. As shown in Fig. 6A, collagen I expression was higher in the cells subjected to flow stimulus than that of the static control. When the flow direction coincided with the fiber direction (0°) with a peak shear stress of 1 dyne/cm^2^, collagen I expression increased more dramatically than any other condition (Fig. 6Avii). Collagen II expression increased as the angle varied from 0° to 90°. The cells exposed to a peak shear stress of 1 dyne/cm^2^ in particular exhibited a dramatic increase in collagen II expression under the perpendicular flow condition (Fig. 6Bix). To detect the secretion of sulfated proteoglycans in the cells, the samples were stained with Alcian blue. The samples under the 45° and 90° conditions exhibited a strong blue stain, which indicated higher sulfated proteoglycan secretion by these cells (Fig. 6Cv–vi and viii–ix).

**Figure 7:**
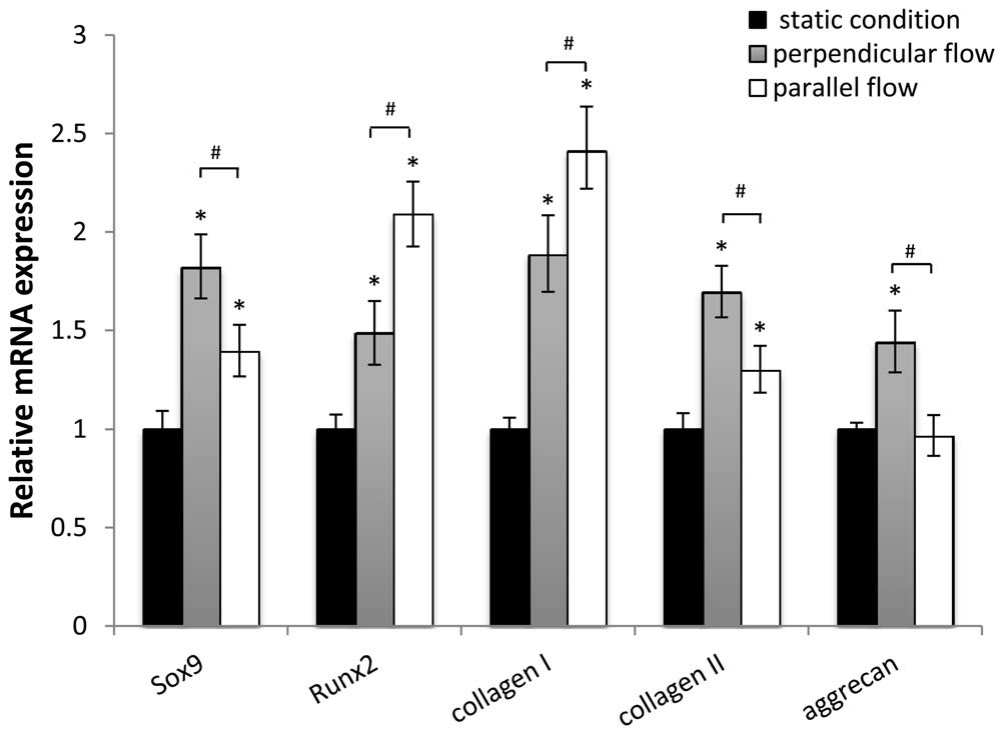
Fibrochondrogenesis-related gene expression in MSCs. Sox9, Runx2, collagen I, collagen II, and aggrecan gene expression was analyzed after 4 weeks of differentiation under perpendicular flow (90°) or parallel flow (0°) with a shear stress of 1 dyne/cm^2^. Data are presented as mean ± SEM. *p<0.05, flow vs. static condition. ^#^ *p*<0.05, perpendicular flow vs. parallel flow.

To further confirm the effects of flow and aligned nanofibers of multiple angles on MSC fibrochondrogenesis, the expression of fibrochondrogenic genes was detected by real-time RT-PCR for samples under static and dynamic conditions (1 dyne/cm^2^), including flow parallel or perpendicular to the fiber direction. Consistent with our immunostaining results, the experimental conditions exhibited dramatic effects on the mRNA expression of fibrochondrogenic markers. As shown in Fig. 7, collagen I and collagen II gene expression was upregulated significantly under both perpendicular and parallel flow conditions compared to the static condition. A significant increase in aggrecan expression was observed under the perpendicular flow condition. The expression of transcription factor Sox9, an early marker of chondrogenic differentiation [38], also increased significantly under both perpendicular and parallel flow conditions compared to the static condition. Similarly, the expression of transcription factor Runx2, which is involved in bone and cartilage development and maintenance [39], was significantly higher in flow conditions compared to the static condition. The cells under the perpendicular flow condition exhibited a significantly higher expression of Sox9, collagen II, and aggrecan, but a significantly lower expression of Runx2 and collagen I compared to those under the parallel flow condition.

### The Role of ROCK and YAP/TAZ in MSCs Fibrochondrogenesis

To elucidate the role of ROCK and YAP/TAZ in MSCs fibrochondrogenesis induced by mechanotransduction, RhoA/ROCK pathway was inhibited and YAP/TAZ expression was knockdown. Since the perpendicular flow condition with a shear stress of 1 dyne/cm^2^ had distinct effects on cell morphology and fibrochondrogenesis, cells on aligned nanofibrous scaffolds under this and static conditions were employed for further investigation. Meanwhile, cells untreated by ROCK inhibitor or YAP/TAZ knockdown were induced differentiation for both perfusion culture and static culture.

**Figure 8:**
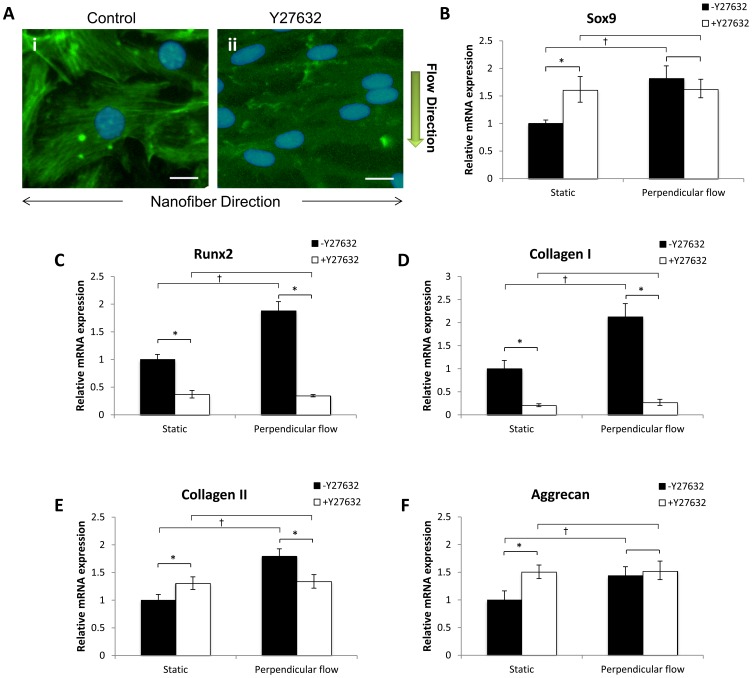
ROCK is involved in fibrochondrogenesis of MSCs. A: Immunofluorescence staining for f-acin (green) and DAPI (blue) after MSCs treated (ii) or untreated (i) with the ROCK inhibitor Y27632 under perpendicular flow stimulus for 6 h. Scale bar: 20 µm. B–F: Fibrochondrogenesis-related gene expression of Sox9 (B), Runx2 (C), collagen I (D), collagen II (E), and aggrecan (F) was analyzed after 4 weeks of differentiation under perpendicular flow with a peak shear stress of 1 dyne/cm^2^. Data were presented as mean ± SEM. *p<0.01, cell treated with Y27632 vs. untreated with Y27632.^ †^ *p*<0.05 perpendicular flow vs. static for Y27632-untreated group. ^#^ *p*<0.05, perpendicular flow vs. static for Y27632-treated group.

After MSCs were treated with ROCK inhibitor Y27632 under dynamic culture for 6 h, we noticed the lack of actin fibril organization in the cells, and no alterations in cell morphology. Meanwhile, the cells of the untreated group had relatively round nuclei and an outspread cell shape (Fig. 8Ai). These results indicated that Rho-kinase inhibition abrogates shear-induced effects on MSC shape (Fig. 8Aii). When Y27632 was added during fibrochondrogenic differentiation under the static condition, the expression levels of Sox9, collagen II and aggrecan were higher than those of the control group (Fig. 8B, E, F). However, there was a significant decrease in Runx2 and collagen I expression (Fig. 8C, D). In contrast, when the cells were exposed to perpendicular flow, Y27632 did not enhance mRNA expression of Sox9 and aggrecan, and even resulted in a significant decrease of collagen II expression compared with the untreated group (Fig. 8B, E, F). Furthermore, Y27632 abrogated flow-induced Runx2 and collagen I expression and caused a significant decrease, indicating that the activation of RhoA/ROCK pathway is necessary for the expression of Runx2 and collagen I (Fig. 8C, D).

**Figure 9:**
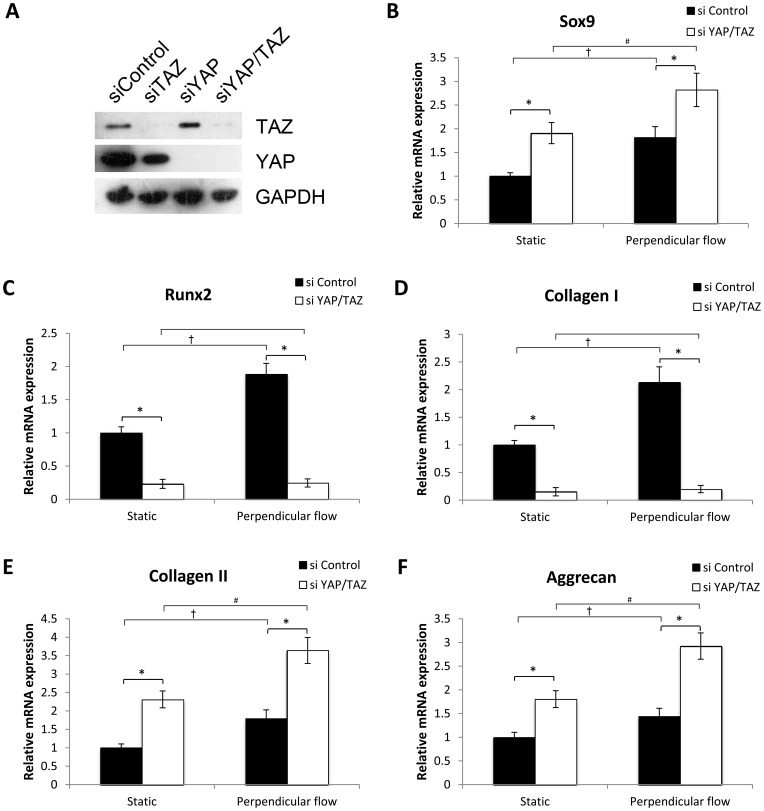
YAP/TAZ is involved in fibrochondrogenesis of MSCs. A: MSCs were transfected with siYAP and siTAZ, and efficient knockdown of the endogenous proteins were analyzed by Western blot analysis. B–F: Fibrochondrogenesis-related gene expression of Sox9 (B), Runx2 (C), collagen I (D), collagen II (E), and aggrecan (F) was analyzed after 4 weeks of differentiation under perpendicular flow with a peak shear stress of 1 dyne/cm^2^. Data were presented as mean ± SEM. *p<0.01, siYAP/TAZ vs. sicontrol. ^†^ *p*<0.05 perpendicular flow vs. static for sicontrol group. ^#^ *p*<0.05, perpendicular flow vs. static for siYAP/TAZ group.

Western blot analysis showed that siYAP and siTAZ led to efficient knockdown of the endogenous proteins (Fig. 9A). In the static condition, YAP/TAZ knockdown enhanced Sox9 expression significantly compared to the control group (Fig. 9B). Similar results were observed for collagen II and aggrecan expression (Fig. 9E, F). Strikingly, compared with the control, YAP/TAZ knockdown elicited a significant increase in the expression of Sox9, collagen II, and aggrecan under the same perpendicular flow condition, which was distinct from that of ROCK inhibition. In addition, the cells subjected to flow stimulus or static exhibited different expression of chondrogenic markers in response to YAP/TAZ knockdown, as we observed significant increase in Sox9, collagen II and aggrecan expression under the perpendicular flow condition in both siControl and siYAP/TAZ transfected cells (Fig. 9B, E, F). The perpendicular flow significantly enhanced Runx2 and collagen I expression in the siControl transfected cells, but there was no difference between static and perpendicular flow conditions in siYAP/TAZ transfected cells. Regardless of flow stimulus or static, YAP/TAZ knockdown completely repressed the induction of Runx2 and collagen I (Fig. 9C, D), suggesting that YAP/TAZ is necessary for Runx2 and collagen I expression. Taken altogether, these data indicate that YAP/TAZ upregulates Runx2 and collagen I expression, and downregulates Sox9, collagen II, and aggrecan expression.

## Discussion

In this study, we first evaluated MSC morphology and fibrochondrogenesis in response to a biomimetic microenvironment of fibrocartilage that integrated both aligned nanofibers and flow stimulus of multiple angles simultaneously. The results suggest that oscillatory flow stimulus is beneficial to fibrochondrogenesis. In particular, the flow direction perpendicular to aligned nanofibers resulted in a fibrochondrogenic phenotype, while parallel flow led to a fibroblastic phenotype with the high level of collagen I expression. These striking differences suggest that the angle between perfusion flow and aligned nanofibers plays a significant role in regulating MSC fibrochondrogenic differentiation.

Local nanotopographical cues and different angles of fluid flow stimulus have considerable impact on the variation of cytoskeletal and nuclear morphology according to their differentiation fate. The spindle-shaped cells of the outer meniscus that maintain a fibrous extracellular matrix rich in collagen I are described as fibroblast-like cells. The round, inner meniscus cells that produce both collagen I and II are described as fibrochondrocytes or chondrocyte-like cells [11]. It has been shown that the geometric shape of a cell is a regulator of stem cell fate. For example, adipogenic commitment is facilitated in a round cell, but osteogenesis is promoted in an elongated and flattened shape [18], [40], [41]. For chondrogenesis, a more rounded, spheroidal cell shape can enhance chondrocyte-related gene and protein expression [42], [43]. Similarly, a more rounded nuclear shape is associated with the expression of chondrogenesis-related markers [43]. In this study, the varying mechanical cues exerted by different angles of fluid flow regulated a fibrochondrogenic or fibroblastic fate. As the angle increased from 0 to 90 degree, the combined effects of the delivery of shear flow-induced and nanotopography-induced cues on cellular morphology and fibrochondrogenic markers made a significant change, although the flow-induced shear stress ranging from 0.01 to 1 dyne/cm^2^ was not enough to alter the direction of the cytoskeleton. Unlike previously reported methods that controlled cell shape using a micropatterning technique, the cellular and nuclear shape were determined by the local mechanical microenvironment in our study. The use of fibrin or gelatin as a scaffold could facilitate cell attachment and an outspread shape under chondrogenic culture conditions, resulting in a fibrochondrogenic phenotype [44]. Thus, it appears that an outspread shape is associated with fibrochondrogenesis during chondrogenic differentiation. Our findings are consistent with previous study showing that increased collagen I expression was dependent on an increase in NAR of cells on aligned nanofibrous scaffolds [45]. In addition, we found that oscillatory flow as a mechanical cue acted synergistically to enhance fibrochondrogenesis-related marker expression, which may explain the noteworthy effect of fluid flow on fibrochondrogenic differentiation of MSCs.

Essentially, the changes in MSCs fibrochondrogenic marker expression due to the mechanical environment are modulated through mechanotransduction systems. It is well established that RhoA/ROCK pathway, a key regulator of the actin cytoskeleton, has the potential to regulate stem cell fate via intrinsic mechanisms [7], [18], [20], [22]. Recently, the role of transcriptional regulators YAP/TAZ as downstream elements in mechanotransduction has been demonstrated [25]. Notably, YAP/TAZ activity requires Rho GTPase activity and tension on the actomyosin cytoskeleton, and has similar effects to RhoA/ROCK in regulating MSC osteogenic or adipogenic fate. To elucidate whether the fibrochondroinductive effect of mechanical microenvironment are mediated by RhoA/ROCK pathway and YAP/TAZ, we inhibited RhoA/ROCK pathway or knockdown YAP/TAZ expression in MSCs. Our results suggest that RhoA/ROCK pathway and tension on the actin cytoskeleton are required for MSCs to perceive and respond to their external mechanical cues for fibrochondrogenic differentiation. Furthermore, YAP/TAZ knockdown in our biomimetic environment led to chondrocyte-like gene expression, but was antagonistic to fibroblast-like gene expression. Under the static condition, YAP/TAZ knockdown and ROCK inhibition both repressed collagen I and Runx2 expression but enhanced chondrocyte-specific marker expression. In contrast, under the perpendicular flow condition, YAP/TAZ knockdown and ROCK inhibition exhibited profoundly different effects: YAP/TAZ knockdown significantly upregulated chondrocyte-specific markers, while ROCK inhibition did not. A possible explanation for this difference is that the ability of the cells to sense and respond to mechanical cues is abrogated by ROCK inhibition, but the depletion of YAP/TAZ could not block other mechanotransduction pathways. Consequently, the cells could still perceive the mechanical microenvironment through the actin cytoskeleton and RhoA pathway.

Fibrochondrogenic differentiation has important significance for regenerative medicine, especially in the application in repairing injured fibrocartilage, such as the meniscus of the knee, temporomandibular joint disc, and intervertebral disc [46]. In this study, using microfabrication techniques we achieved multi-angle fluid flow stimuli in a micron-scale platform integrated with aligned nanofibers to mimic the native fibrocartilage microenvironment. The design of biomaterials with multi-angle mechanical stimuli using microfluidic technology represents a valid approach to modulate the stem cell niche. Recent advances in micro- and nanofabrication techniques have enabled the design of more biomimetic biomaterials [47], but a dynamic culture platform seems conducive to advanced studies of cell-substrate interactions. Nanopatterns and fluid flow can be employed independently or in concert to manipulate cell biological behaviour [48], [49]. However, it has been challenging to integrate nanofibers into the lab-on-chip. In this study, we developed a simple and low-cost way to rapidly fabricate microfluidic devices integrated with nanofibers. Compared with micro- and nanopatterning methods, nanofibers better mimic native tissue architecture, serving as an accommodative milieu similar to 3D ECM. Moreover, the nanofibers can be further functionalized by attaching bioactive molecules such as proteins and growth factors. Thus, microfluidic technology and biomaterials and their associated micro- and nanofabrication schemes have enabled the design of artificial microenvironments that may offer novel ways to direct stem cell fate.

Some limitations of this study should be pointed out, such as the inability to create large-scale functional tissue constructs and the lack of in-depth study of the mechanotransduction mechanism. In addition, the alignment and deformation of the nuclei can induce the changes of gene and protein expression [50], and is also related to the lineage commitment of stem cells [51]. Whether the deformation of nuclei exerts direct effects on the transcriptional mechanotransduction regulators such as YAP and TAZ require further investigation.

### Conclusions

We presented a novel microfluidic perfusion device that integrated nanotopography and flow stimulus of varying orientation simultaneously. Our results suggest that the angle between perfusion flow and aligned nanofibers play a significant role in regulating fibrochondrogenic differentiation of MSCs, which is mediated by both RhoA/ROCK pathway and YAP/TAZ. Further in-depth understanding of the dynamics of stem cell niches and their effects on mechanotransduction will help us tune the fate of stem cells and optimize their application via the design of novel biomaterials and microdevices.
